# Production of Kudzu Starch Gels with Superior Mechanical and Rheological Properties through Submerged Ethanol Exposure and Implications for In Vitro Digestion

**DOI:** 10.3390/foods12213992

**Published:** 2023-10-31

**Authors:** John-Nelson Ekumah, Xu Han, Qiufang Liang, Lixin Kang, Benxi Wei, Arif Rashid, Muhammad Safiullah Virk, Abdul Qayum, Selorm Yao-Say Solomon Adade, Nana Adwoa Nkuma Johnson, Xiaofeng Ren

**Affiliations:** 1 Department of Food Science and Engineering, School of Food and Biological Engineering, Jiangsu University, 301 Xuefu Road, Zhenjiang 202013, China; 2 Department of Nutrition and Food Science, College of Basic and Applied Sciences, University of Ghana, Legon P.O. Box LG 134, Ghana; 3 Institute of Food Physical Processing, Jiangsu University, 301 Xuefu Road, Zhenjiang 212013, China

**Keywords:** kudzu starch, gels, mechanical properties, rheological properties, in vitro digestion

## Abstract

Producing starch gels with superior mechanical attributes remains a challenging pursuit. This research sought to develop a simple method using ethanol exposure to produce robust starch gels. The gels’ mechanical properties, rheology, structural characteristics, and digestion were assessed through textural, rheological, structural, and in vitro digestion analyses. Our investigation revealed an improvement in the gel’s strength from 62.22 to178.82 g. The thermal transitions were accelerated when ethanol was elevated. The exposure to ethanol resulted in a reduction in syneresis from 11% to 9.5% over a period of 6 h, with noticeable changes in size and color. Rheologically, the dominating storage modulus and tan delta (<0.55) emphasized the gel’s improved elasticity. X-ray analysis showed stable B- and V-type patterns after ethanol exposure, with relative crystallinity increasing to 7.9%. Digestibility revealed an ethanol-induced resistance, with resistant starch increasing from 1.87 to 8.73%. In general, the exposure to ethanol played a crucial role in enhancing the mechanical characteristics of kudzu starch gels while simultaneously preserving higher levels of resistant starch fractions. These findings have wide-ranging implications in the fields of confectioneries, desserts, beverages, and pharmaceuticals, underscoring the extensive academic and industrial importance of this study.

## 1. Introduction

Hydrogels are characterized as polymer networks that are hydrophilic and three-dimensional in nature. They possess a notable capacity to absorb and retain significant amounts of water or biological fluids. Their high water retention capacity, coupled with their inherent softness and flexibility, opens up numerous potential applications across various fields [1,2]. Within the food industry, hydrogels encompass a wide range of substances that span from fully developed products to unprocessed components utilized in the production of novel food derivatives [1]. In the specific context of confectioneries (gummy candies), beverages (bubble teas), and gel-based desserts (panna cottas), the pressing need for hydrogels with consistent rheological and textural properties has become paramount to ensure optimal product quality and consumer satisfaction. Moreover, such hydrogels effectively encapsulate active ingredients, including antimicrobials and antioxidants, further elevating the general quality of the products [3,4]. 

Despite their promise, hydrogels’ poor mechanical and rheological properties frequently hinder their widespread industrial application. This shortcoming is generally linked to imperfections in the cross-linking process, leading to an inconsistent structural network. Moreover, inadequate energy dissipation mechanisms contribute to their poor resistance to mechanical stress and strain [5,6]. Thus, the need for the development of hydrogels with enhanced mechanical properties for diverse applications, including food and non-food products, is evident.

Scientists have been innovative in addressing these challenges, exploring structural designs and energy dissipation systems and incorporating techniques such as post-gelation treatments [7,8]. Post-gelation exposure to solutions containing ions or cross-linking agents has emerged as a relatively simple and efficient approach. The application of this treatment results in the initiation of dehydration, reorganization of the structure, and transfer of constituents within the hydrogel, ultimately resulting in the formation of a resilient network. The aforementioned procedure has the potential to increase the crosslinking density, thereby resulting in enhanced mechanical and rheological properties [2,8].

Ethanol, a solvent that is semi-polar, water-miscible, and innocuous, presents an intriguing solution following the process of gelation. The dehydration and structural reconfiguration capabilities have the potential to enhance the internal molecular strength of the hydrogel [2,9]. Nevertheless, the effectiveness of this procedure relies on specific factors including the concentration of ethanol, duration of exposure, and the specific natural biomolecules being targeted. Notably, higher ethanol concentrations induce changes in solubility and increase tensile strength in hydrogels. It is suggested that the molecular exchange in gels in the presence of ethanol can enhance the internal network chains, inducing retrogradation and thus improving the mechanical and rheological properties of hydrogels [9,10].

There has been a recent focus on starch, an essential polysaccharide found in biopolymer-based hydrogels, due to its biocompatibility, biodegradability, and significant biological functionality [3,11]. In particular, kudzu (*Pueraria lobata*) starch [12], with its distinctive characteristics, shows promising gel properties [13]. However, like many physically synthesized gels, kudzu starch gels commonly lack the desired mechanical strength and exhibit suboptimal rheological performance. The aforementioned properties could be greatly improved through the utilization of the innovative and expeditious post-gelation ethanol-exposure technique [1,8].

Nonetheless, the effects of alcohol-exposure techniques go beyond the mere physical characteristics of the gel. Potential health implications may arise from alterations in starch digestibility, specifically in relation to blood glucose levels, glycemic response, gut health, and weight management [14]. Therefore, the implications of ethanol exposure on the in vitro digestion characteristics of kudzu starch gels are also worth investigation. Currently, there is a lack of documented research examining the effects of ethanol exposure as a gel-improving approach on the mechanical, rheological, and in vitro digestion characteristics of kudzu starch gels. The objective of this study is, therefore, to evaluate the effects of post-gelation ethanol treatment on the mechanical properties, rheology, and the in vitro digestibility of kudzu starch gels. The findings from this research could enhance the value of kudzu starch gel and facilitate its broader application within the food industry specifically, in confectioneries, gel-based desserts, beverages, and edible nutritional pharmaceuticals.

## 2. Materials and Methods

### 2.1. Materials

Kudzu roots was procured from Xi’an Nature Choice Co., Ltd. (Tianjin, China). Ethanol, hydrochloric acid (HCl), and sodium hydroxide (NaOH) were also obtained from Sinopharm Chemical Reagent Co., Ltd. (Shanghai, China). Salivary amylase, Pancreatic α-amylase, and 3,5-dinitrosalicylic acid (DNS) were also supplied by Sigma-Aldrich (Shanghai, China). All other chemicals were of analytical grade with >99% purity.

### 2.2. Kudzu Starch Gel Formation

Kudzu starch was extracted from the roots following a conventional starch extraction method [14]. A 25% (*w*/*v*) starch suspension was prepared from the extracted starch and gelatinized at 90 °C for 30 min, with regular stirring. The resultant gel was poured into a 10.0 mL cylindrical mold and allowed to set at room temperature. Subsequently, the gels were immersed in ethanol solutions of varying concentrations (30%, 60%, 80%, and 100%). The set-up of gel immersed in alcohol solution was allowed to stand at room temperature over varying periods of 2, 4, and 6 h for each concentration. For comparison, a control sample was prepared with the same gelling approach without ethanol exposure but kept at 4 °C for 12 h. After the designated periods, samples were appropriately removed and kept for further analysis.

### 2.3. Gel Syneresis, Size, and Color Characteristics

Sample gels after ethanol exposure were stored at 4 °C for 24 h, thawed, and centrifuged at 5000× *g* for 15 min. The amount of water released from the gel was weighed and used to calculate syneresis degree, expressed as a percentage of the initial gel weight [15]. The size of the gels exposed to ethanol were measured and reported as volume (cm^3^) using Equation (1). The color of gels was also assessed following the method used by Ekumah et al. [16]. The C.I.E.LAB color parameters (lightness (L*), redness (a*) and yellowness (b*) of the samples) were measured using the HunterLab ColorQuest XE Spectrophotometer (Hunter Associates Laboratory, Reston, VA, USA).

(1) $$\mathrm{Gel}\;\mathrm{volume}\;\left({\mathrm{cm}}^{3}\right)={\pi r}^{2}\;h$$

### 2.4. Mechanical Properties

The determination of the mechanical properties of the gels were achieved using a texture profile analyzer (TA.XT plus, StableMicro Systems, Surrey, UK) equipped with a 0.5 inch probe (*p* 0.5) after calibrating it with a 1 kg load. The test speed and compression strain were set at 1 mm/s and 75%, respectively. The hardness (g), springiness, chewiness, and resilience data were then recorded [9].

### 2.5. Rheological Properties

The starch gel’s rheological characteristics were evaluated using a DHR-1 rotation rheometer (TA instruments-Waters, Shanghai, China), equipped with a 40 mm diameter parallel plate and a gap of 1.0 mm. The apparent viscosity was studied across a shear rate ranging from 0 to 300 s^−1^. Measurements were taken for the elastic modulus (G′), viscous modulus (G″), and loss tangent (Tan δ) based on the angular frequency (ω) spanning from 0.1 to 100 rad/s, ensuring a 2% strain within the linear viscoelastic domain. Modeling of the rheological properties was achieved by fitting the curve to the power model, Equation (2) [17].

(2) $$ɳ=K\gamma^{n-1}$$

From the model, ɳ, *K*, *n*, and γ denote viscosity (mPa.s), flow consistency coefficient, flow behavior index, and shear rate (s^−1^), respectively.

### 2.6. Scanning Electron Microscopy (SEM)

The morphological characteristics and microstructure of starch gels were evaluated using scanning electron microscopy (SEM) according to Ahmad et al. [18]. Freeze-dried starch gel samples were loaded into an aluminum stub using double-sided adhesive carbon tape. The sample photographs were taken using a JSM-7001F SEM apparatus (JEOL USA Inc., Peabody, MA, USA) at an accelerating potential of 15 kV.

### 2.7. X-ray Diffraction (XRD)

The X-ray diffractometer (D2 PHASER, Bruker, Germany) at 40 kV and 30 mA with Cu-Ka radiations was used to determine the crystallographic structural characteristics of the starch gel samples. The X-ray diffraction profile was obtained for 2θ from 5° to 40° with a step size of 0.02/min. The Origin software (Version 19.0, Microcal Inc., Northampton, MA, USA) integrated the amorphous and crystalline areas, while the relative crystallinity was estimated from Equation (3) as described by Zhang et al. [19].

(3) $$Crystallinity=\frac{A_{C}}{A_{C}+A_{a}}\times 100$$

### 2.8. Fourier Transform Infrared Spectroscopy (FTIR) Analysis

The chemical and structural configuration of sample starch gels was evaluated using attenuated total reflectance–Fourier transform infrared spectroscopy (ATR-FTIR) (Nicolet iS10, Thermo Electron Inc., Waltham, MA, USA) following procedures reported by Wu et al. [20]. In summary, the freeze-dried powdered starch gel samples were directly loaded onto the detection platform and scanned at a resolution of 4 cm^−1^. The spectra ranging from 4000 to 400 cm^−1^ were recorded three times for each sample in transmission mode and evaluated by Origin software (Version 19.0, Microcal Inc., Northampton, MA, USA).

### 2.9. Differential Scanning Calorimetry (DSC)

The thermal properties of the starch gels were determined by differential scanning calorimetry (DSC) using a differential scanning calorimeter (DSC-60, Shimadzu, Singapore) as per the method described by Ahmad et al. [18]. A sample of dried starch gel (3 mg) was weighed in the aluminum pans and mixed with water in a ratio of 1:3. The pans were sealed and allowed to stand for 12 h at 4 °C for moisture equilibration. The samples were then scanned from 25 to 120 °C at 10 °C/min. The equipment was calibrated with indium, and an empty sealed pan was used as a reference for all experiments. The onset temperature (To), peak temperature (Tp), conclusion temperature (Tc), and gelatinization temperature range (Tc-To) were noted from the graphs. The enthalpy of gelatinization (ΔH) was estimated by integrating the area between the thermograms and a baseline under the peak and reported as joules per gram of dry starch gels.

### 2.10. In Vitro Digestibility

The in vitro digestion of starch gels was achieved following the INFOGEST static in vitro simulated gastrointestinal digestion method [21]. Briefly, a 5 g starch gel was mixed with 5 g Simulated Salivary Fluid (SSF), Table 1; 75 U/mL salivary amylase was achieved by adding 3 mL of the enzyme and pH was adjusted to 7 with NaOH (1.0 M). The mixture was then incubated at 37 °C for 2 min amidst agitation. The resultant oral bolus was subsequently mixed with an equal volume of Simulated Gastric Fluid (SGF) (Table 1) to attain a final ratio of 1:1 (*v*/*v*). The pH was adjusted to 3.0 with HCl (1.0 M) and incubated at 37 °C for 2 h with agitation. Furthermore, the chyme from the gastric digestion was mixed with an equal volume (1:1 *v*/*v*) of SIF preheated at 37 °C. The pH was adjusted to 7.0 by adding NaOH (1.0 M). Bile salt (10 mM) was added, followed by pancreatic alpha-amylase (200 U/mL). The set-up was incubated for 2 h at 37 °C in a shaking incubator. Finally, the digested product was centrifuged at 14,000× *g* for 15 min to collect the transparent supernatant for DNS analysis, to determine the digestible, as well as the pellet for the determination of resistant starch proportion of the gels.

### 2.11. Statistical Analysis

All samples were analyzed in triplicate, and the results were presented as means and standard deviations. A one-way analysis of variance (ANOVA) was used to compare means while Tukey test was used to study the significant differences between the mean values at *p* < 0.05. Pearson’s correlation analysis was also used to find the correlation between the factors evaluated. All the analyses and graphs were achieved using Origin (Version 2019, Microcal Inc., Northampton, MA, USA).

## 3. Results and Discussion

### 3.1. The Textural Characteristics of Kudzu Starch Gels

The control exhibited the lowest values for all measured parameters, as shown in Table 2. The study additionally demonstrated that exposure to ethanol, even at low concentrations, resulted in a statistically significant increase in the hardness of the gels, regardless of the duration of exposure [2]. However, as the concentration of ethanol increased, the hardness of the gels increased across all exposure durations, with the 100% ethanol at 6 h marking the highest at 178.82 g. This implies that ethanol-induced changes in the structure of starch resulted in increased resistance, likely due to a mechanism involving the removal of water through ethanol dehydration [7,8]. Similarly, with an increase in ethanol concentrations, there was a corresponding increase in springiness across all parameters. The sample subjected to 100% ethanol concentration over a 6 h interval reached a springiness of 1.01. This data suggests an augmentation in the molecular elasticity of the starch gel matrix post ethanol treatment, enhancing its ability to recover after deformation. Such enhanced recovery properties can have a profound advantage in foods, especially in gummy confectioneries and food nutrient delivery agents, driving innovation in texture, consistency, and functional benefits. Furthermore, the resilience exhibited similar trends as those observed in springiness. The observation indicates that kudzu starch gels treated with ethanol exhibit enhanced energy dissipation properties, thereby reducing the occurrence of permanent deformation when subjected to external forces. This characteristic is lacking in numerous natural biopolymer hydrogels [5,8,10].

Furthermore, the patterns displayed in resilience closely mirrored those seen in springiness, possibly due to their interconnectivity supported by a positive correlation (r = 0.795). From the control (61.31), the value peaked at 91.15 with 100% ethanol for 6 h, indicating superior energy dissipation in ethanol-treated gels as observed in a similar study by Sun et al. [2]. With the enhancement in the ethanol-treated kudzu starch gel’s energy dissipation, they appear to be more durable and will resist degradation when packaged and stored, which could extend shelf life due to enhanced structural stability. The chewiness exhibited a rise in value with ethanol concentration, especially over extended durations. The 6 h, 100% ethanol sample reached 79.89, corroborating the report by Lin et al. [6]. Utilizing these gels as kudzu gel cubes and pearls in beverages like bubble tea and whole milk becomes notably ideal, delivering a conspicuously chewy mouthfeel. The substantial implication here is the possible increase in resistance to masticatory forces. The evident increase in chewiness and resultant resistance to forces imply that the mouth-feel experience in beverages and snacks will remain consistent throughout consumption. This uniformity from the first to the last bite can elevate the overall eating and drinking experience, making it particularly pertinent for the food and beverage industry. Consequently, the data presented in Table 2 clearly demonstrated that kudzu starch gel properties were influenced by both ethanol concentration and exposure time in a synergistic manner. Specifically, longer durations of exposure amplified the impact of ethanol on the kudzu starch gels.

### 3.2. Thermal Properties of the Kudzu Gels

Table 3 shows the effects of the ethanol concentrations and exposure time on the thermal properties of the starch gels. The data in the table reflects significant variances in thermal properties with changing ethanol concentrations and exposure time. A clear trend observed is the fluctuation of onset, peak, and conclusion temperatures (T_o_, T_p_, T_c_) and gelatinization enthalpy (ΔHg) as ethanol concentration and exposure time changed. This implies that the interaction of intrinsic factors, such as slight variations in crystallinity (Table 3) and the reconfiguration of the gel network, played a role in the transition, in addition to the concentration of ethanol and the duration of exposure [22].

For control samples (0% ethanol), the onset temperature (T_o_) was 45.32 °C, peak temperature (T_p_) was 53.92 °C, and conclusion temperature (T_c_) was 66.92 °C. This finding corroborates the findings of Sun et al. [2], which propose that untreated physically cross-linked gels possess advantageous properties for the delivery of heat-sensitive bioactive compounds [23]. Moreover, with an increase in ethanol concentration, the treated samples exhibited fluctuating onset, peak, and conclusion temperatures, without a discernible trend towards an increase. The T_c_-T_o_ narrowed; notably, the 100% ethanol sample at 6 h (16.53–53.53°C) was 6.52 °C, the smallest value recorded, indicating that the thermal transition became quicker as ethanol concentration increased. This observation may indicate a heightened rate of thermal transition at elevated ethanol concentrations. The aforementioned attribute is highly sought after in the industrial sector, as it serves as a crucial factor in determining the level of energy utilized during the ethanol-based kudzu gel processing [2,10,12].

Furthermore, the gelatinization enthalpy (ΔHg) varied with changes in ethanol concentration and exposure time. The highest ΔHg was observed for 100% ethanol concentration at 2 h (2.97 J/g). This could be attributed to the improved cohesion and internal strength conferred on the gel with ethanol exposure irrespective of the exposure time [5,6]. This high ΔHg compared to the control signifies a robust matrix directly influencing gel’s reactions to external stimuli. Essentially, a starch gel of this nature demonstrates enhanced stability against challenges such as thermal fluctuations, applied pressure, and the presence of chemical additives [3]. The results of the study indicate that the thermal properties of starch gels are notably affected by both the concentration of ethanol and the duration of exposure. These findings have important implications for the stability and texture of the gel. 

### 3.3. Gel Syneresis, Size, and Colorimetric Properties

Gel production that deviates from the traditional production route towards improving its quality characteristics has the potential to affect the syneresis, gel size, color, and general appearance. As presented in Table 3, the analysis of syneresis in starch gels exposed to varying concentrations of ethanol over different time intervals provides an understanding of ethanol’s role as a modulating agent in starch-based systems [15,24]. Starting with the control group, a natural decline in syneresis from 11% to 9.5% over 6 h suggests that the gel matrix undergoes dehydration and maturation, leading to better matrix stability [24,25]. The observed decrease in gel properties was found to be more pronounced as the ethanol concentration increased, providing further evidence for the influence of soaking duration on the enhancement of cross-linking properties in the gel [26]. Moreover, the data demonstrates a consistent reduction in syneresis with increasing ethanol concentration. This suggests that ethanol may act as a plasticizer, enhancing the mobility of polymer chains and consequently improving the capacity to dissipate energy when subjected to stress [15,27]. This observation is in line with previous studies which indicate that solvents can alter the swelling behavior of biopolymer networks, although the specifics of rate and degree of syneresis reduction appear to be novel and warrant further investigation [2,8]. These findings may hold substantial significance for industries that require gel matrixes with a stable texture and low moisture content. This is particularly relevant for confectioneries and certain pharmaceutical applications designed for the delivery of essential minerals and nutrients [1,24].

**Figure 1:**
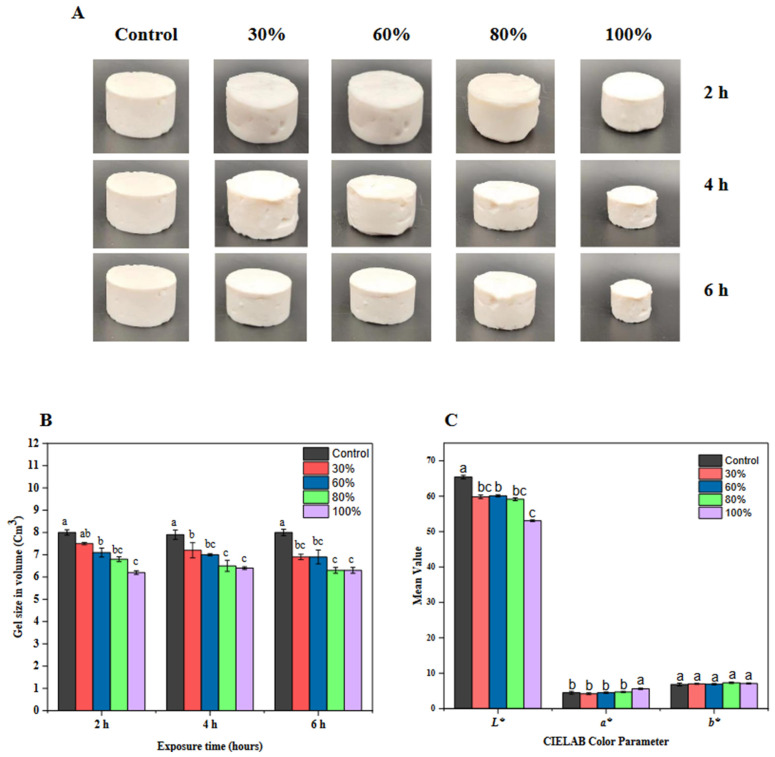
(**A**) Physical appearance of kudzu starch gel exposed to varying concentrations of ethanol and exposure time. (**B**) Gel size of kudzu starch gel exposed to varying concentrations of ethanol for 2, 4, and 6 h. (**C**) CIELAB color parameter of kudzu starch gels at different concentrations at the end of the exposure time. Lowercase alphabets (a, b, c) denote statistical differences. Bars with different alphabets within a cluster are significantly different (*p* < 0.05).

In Figure 1, a pictorial representation of gel size (A), the measured gel sizes in volume (B), and the mean color values of the CIELAB color parameters were presented. According to Figure 1A,B, gel size in volume (cm^3^) decreased as ethanol concentration increased. Within 2 h, gel size shrank from 8.0 cm^3^ in controls to 6.3 cm^3^ at 100% ethanol. By 6 h, it compressed further to 6.2 cm^3^. Ethanol’s solvent nature possibly reconfigured the gel network, causing this shrinkage. This aligns with prior research demonstrating solvents’ effects on gel structures [28,29,30]. In addition, it has been shown that ethanol has the potential to initiate syneresis, a phenomenon that is supported by a significant connection (r = 0.746; Table 4) between the occurrence of syneresis and the size of the gel. In terms of color (Figure 1C), the ‘L*’ value decreased with rising ethanol levels, suggesting darkening gels. For instance, the control had ‘L*’ value of 65.4 drops to 53.1 at 100% ethanol. Both ‘*a*’ and ‘*b*’ values fluctuated: the ‘a*’ value increased to 5.6, and ‘b*’ peaked at 7.3 at 80% ethanol, denoting shifts in color. Such color variations could be due to ethanol’s interaction with the gel matrix, a phenomenon seen in food gels [30,31]. Correlations between ‘b*’ and syneresis (r = 0.653) and between gel size and ‘a*’ (r = 0.545) highlight the intertwined nature of these attributes. The manipulation of ethanol concentration and exposure time has a significant impact on the structural and aesthetic characteristics of gels, thereby offering a promising opportunity for optimizing gels in industrial applications.

### 3.4. Rheological Properties

**Figure 2:**
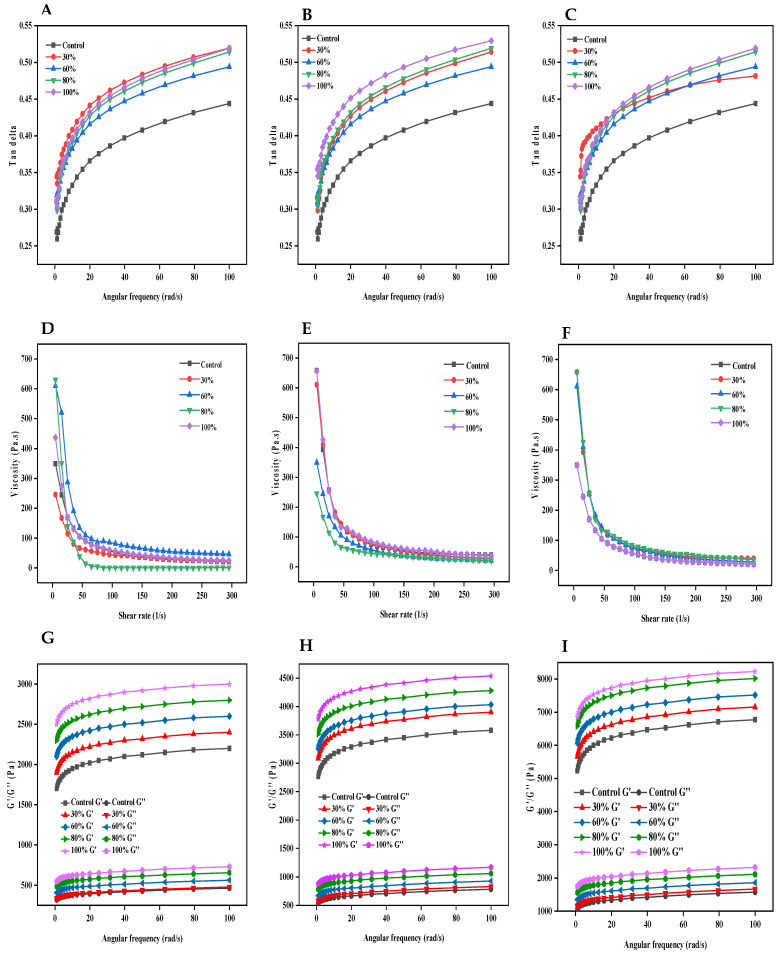
Rheological parameters of ethanol-exposed kudzu starch gel. Tan delta of kudzu starch gel exposed to varying concentrations of ethanol for (**A**) 2 h, (**B**) 4 h, and (**C**) 6 h. Change in viscosity with shear rate of kudzu starch gel exposed to varying concentrations of ethanol for (**D**) 2 h, (**E**) 4 h, and (**F**) 6 h. Storage modulus (G′) and loss modulus (G″) of kudzu starch gel exposed to varying concentrations of ethanol for (**G**) 2 h, (**H**) 4 h, and (**I**) 6 h.

The rheological data presented in Figure 2 showcased variations in rheological parameters, with a clear decline in viscosity as shear rate increases, highlighting the starch gel’s shear-thinning behavior (Figure 2D–F). Roux et al. [29] attribute this to starch molecule realignment under shearing. It could be inferred that ethanol enhances the gel matrix’s fluidity, promoting starch realignment. Over time, ethanol interactions may concentrate the gel, increasing resistance at low shear rates and accentuating shear-thinning at higher rates due to matrix disruption. Furthermore, the power law model was employed to characterize the shear thinning behavior, and the results are detailed in Table 2 with R^2^ > 0.95 for all samples. This could suggest that models could be use in the process optimization and consistent production of mechanically stable kudzu starch gels. Furthermore, the rheological characteristics, particularly related to the viscoelasticity of the kudzu starch gels, reveal their structural and functional dynamics under varied ethanol conditions [2]. Our study’s Tan δ values, consistently below 0.55, signify a predominantly elastic nature. The control’s Tan δ values, which ranged from 0.26 to 0.44, further corroborate this observation. Such low values emphasize and affirm a sturdy and robust gel network. The exposure to ethanol over time, however, introduced a slight plasticizing effect that increased the Tan δ values. Nonetheless, values were still within an appreciable elastic domain [2]. This trend is reminiscent of findings in earlier research, where low Tan δ values have been frequently associated with a robust gel network [8,28]. Nevertheless, the increasing Tan δ values with prolonged ethanol exposure underscore a diminishing elasticity, likely due to ethanol’s interference with the intricate network of the starch gel, reducing its size and elastic nature.

Examining the relationship between the storage modulus (G′) and the loss modulus (G′′) reveals their crucial distinction. A consistent G′ greater than G′′ throughout the experimental angular frequency indicates the gel’s dominant elastic behavior. Interestingly, the elevation of G′ with increasing ethanol concentration and exposure time suggests that, while ethanol disrupts the gel network (as inferred from Tan δ values), it might simultaneously be inducing a structural reorganization or densification in the gel matrix. This behavior, where G′ rises with solvent concentration or exposure time, has been observed in other polysaccharide systems exposed to different solvents [9,28]. For industries, understanding these subtle changes is crucial since modulating ethanol concentration offers a tool to modify the textural properties of starch-based hydrogels. Enhanced gel strength can elevate food texture and appeal while affecting product delivery in pharmaceuticals or cosmetics; thus, our results hold both academic and industrial significance.

### 3.5. Scanning Electron Microscopy

**Figure 3:**
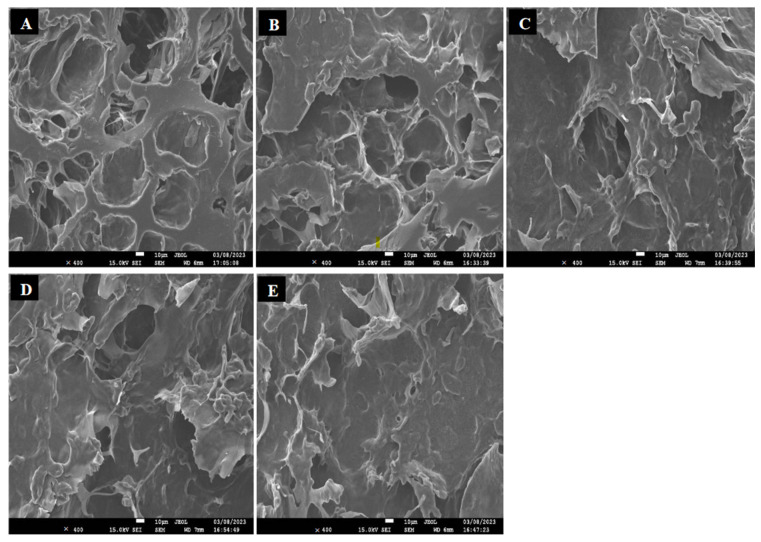
Scanning electron micrograph of kudzu starch gel exposed to varying concentrations of ethanol; (**A**) control, (**B**) 30%, (**C**) 60%, (**D**) 80%, (**E**) 100%.

The Scanning Electron Microscope (SEM) offers an in-depth visualization of the microstructural changes in materials, including starch gels. When kudzu starch gels were exposed to varying concentrations of ethanol and for increasing time duration, several microstructural alterations were observed. Figure 3 shows the SEM images of the internal morphology of the freeze-dried starch gels. All samples exhibited typical porous three-dimensional network structures, with the control gel having the largest and open microstructures, while that of the ethanol-exposed gels reduced with increasing ethanol concentration. The flaky surface of the control gel (Figure 3A) exhibited a loose microstructure with comparably larger air spaces. Post ethanol exposure, however, the pore wall structure of the starch gels became densified with an apparent reduction is pore space (Figure 3B–E). This corresponded with the gel size reduction with ethanol and increasing exposure time. The phenomenon might have been caused by the osmotic pressure of the alcohol, and this might have increased the packing density of the starch polymer chains [8,30,31]. The starch gel pore walls in Figure 3B–D were relatively flat and smooth with decreasing holes as the concentration of ethanol increased. The 100% ethanol exposure (Figure 3E) typically revealed a highly reduced microstructure with smooth surfaces. According to Zheng et al. [8], ethanol dehydrates the gel at high concentration by removing bound and free water, compacting its microstructure and causing it to partially collapse, resulting in smoother surfaces. Additionally, ethanol being polar in nature interacts with starch gel’s network, disrupting and reorganizing the gel matrix, leading to a denser, smoother structure [2,8].

### 3.6. X-ray Diffraction Analysis

**Figure 4:**
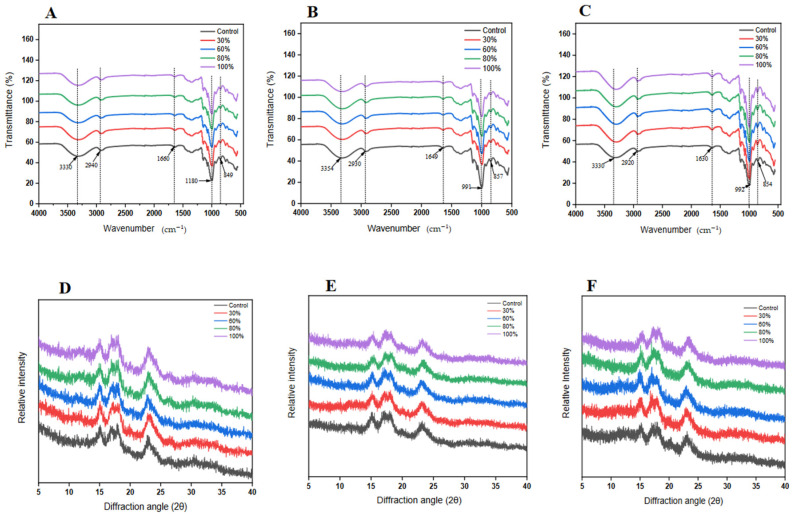
FTIR spectra of kudzu starch gel exposed to varying concentrations of ethanol for (**A**) 2 h, (**B**) 4 h, and (**C**) 6 h. X-ray diffraction pattern of kudzu starch gel exposed to varying concentrations of ethanol for (**D**) 2 h, (**E**) 4 h, and (**F**) 6 h.

The diffractive peak values are significant for understanding the structural changes in materials when exposed to various conditions relying on their A-, B-, or C-type crystallinity pattern [32]. Here, Fig. 4 presents the diffractive peak values of kudzu starch gel with and without ethanol exposure over different time intervals. The gels without ethanol exposure exhibited peaks at 15.33°, 16.75°, and 17.82°, showing the typical diffraction pattern of a B-type structure [9,33,34], with a more hydrophilic configuration typical of root and tuber polysaccharides. In addition, the starch gel had an apparent peak at 23.00°, which exemplifies a typical V-type structure exhibited by macromolecules that has undergone heating and subsequent retrogradation. Therefore, the control starch gel was characterized by a B- and V-type structure. Comparably, the gel samples exposed to ethanol had characteristic peaks like the control at 15.33°, 16.75°, 17.89°, and 23°, thus indicating a typical B- and V-type structure, suggesting both the regular B-type crystallinity and a heat- and water-interaction-driven V-type configuration. Therefore, with the subtle change in peak values, the crystalline type of starch gel did not change after ethanol exposure. This observation in kudzu starch gel aligns with finding in similar solvent-exposed polysaccharide studies [35,36].

The relative crystallinity (Table 3) of the starch gels with and without ethanol exposure was calculated with diffractograms. The crystallinity value of the control kudzu starch gel remained stable at 4.7 across all time points (2, 4, and 6 h), highlighting the consistent crystalline nature of the starch gel in the absence of ethanol. Concurrently, as the ethanol concentration rises, a distinct trend of increasing crystallinity values emerged, with each time frame showing a progressive increase in crystallinity from 30% to 100% ethanol concentration. Additionally, each ethanol concentration demonstrates a time-dependent augmentation in crystallinity from 2 to 6 h. The crystallinity of the non-ethanol-exposed starch gel was the lowest at 4.7%. Starch gels exposed to ethanol showed an increase in crystallinity, rising from 5.2% at 30% ethanol concentration for 2 h to 7.9% at 100% concentration for 6 h, highlighting ethanol’s effectiveness in enhancing starch gel crystallinity. Ethanol tends to disrupt the organized structure of starch molecules, changing their crystalline attributes. The data presented reveals an uptick in crystallinity as ethanol concentration rises, insinuating ethanol’s role in fostering a more regimented starch molecule arrangement in the gel [2,29]. This observation can be potentially attributed to ethanol, as a solvent, initiating partial perturbation of the starch gel’s amorphous sections, thereby amplifying the relative crystalline area. This aligns with several studies illustrating the solvent-induced enhancement of starch crystallinity through amorphous region disruption [30,37,38]. Moreover, the increasing crystallinity with ethanol concentration underscores a pronounced ethanol–starch interaction, possibly due to amplified solvent infiltration disrupting the less-ordered regions [10]. This crystalline transition was not immediate but evolves and stabilizes over time upon sustained ethanol contact.

### 3.7. FTIR Spectra Analysis

The structural configuration of kudzu starch gels after timed ethanol exposure was characterized by FTIR as presented in Figure 4. The figure revealed a high similarity between the timed-ethanol-exposed gels and that of the control. The findings indicate that, while there may be potential changes in the structure of the ethanol-exposed gels, no new compounds were introduced from the ethanol solutions during the specified exposure period [12,39]. This observation corresponds with the results reported by Sun et al. [2] in their study on the enhancement of maize starch gel properties through cross-linking. The consistent spectra provide assurance that any variations in the properties of the gels are not due to the introduction of new chemical entities but a potential reconfiguration of existing bonds or subtle alterations in molecular environments. In this regard, with an increase in exposure time there were negligible subtle changes in the band of relevance to the properties of the gels. The spectra of the gels exhibited broad bands at 3330 cm^−1^ for 2 and 6 h (Figure 4A,C) and at 3354 cm^−1^ for 4 h (Figure 4B). This is indicative of the presence of hydrogen bonding interactions of the hydroxyl (O-H) groups typical of natural polymers composed of amylose and amylopectin (starches). The variability, however, suggests the extent and strength of hydrogen bonds which were largely maintained even after ethanol exposure [40]. This property is significant when considering the gel’s utility in controlled-release systems or encapsulation applications, where the maintenance of the gel structure is imperative. The band within the range of 2920 to 2940 cm^−1^ was ascribed to the stretching of CH_2_ groups associated with starch monomers including glucose. The observed variability may be associated with variations in the duration of exposure. Ethanol, being a polar molecule, has the ability to engage in interactions with starch molecules, leading to alterations in their structure. This is achieved by modifying the surrounding environment of the C-H bonds present in the glucose units, thereby inducing subtle changes over time [35,41]. Again, exhibiting of bands at 1660, 1649, and 1630 cm^−1^ for the 2, 4, and 6 h samples is indicative of the presence of bound water in the gel (water-bending vibration). The phenomenon of time dependence was observed in the fluctuation of wavenumber over time. Specifically, shorter durations were found to be associated with higher wavenumbers, indicating an increase in the amount of bound water present in the starch gels. This increase in bound water content can be attributed to the marginal dehydrating effect caused by prolonged exposure to ethanol. This supports the conclusion drawn by Zhao et al. [14], buttressed by the physical gel appearance (Figure 1A). C-O-C glycosidic linkage stretching typical of polysaccharides was shown at 1180 cm^−1^ while the C-O stretching vibration was shown between 992 and 857 cm^−1^ of the 4 and 6 h ethanol-exposed gel. These FTIR findings not only reaffirmed the molecular intricacies of kudzu starch gels but also emphasized their robustness post ethanol exposure. The subtle distinction observed in the spectra highlights the need for considering such when designing systems that leverage the unique properties of these gels, especially in food, beverages, and edible nutrient delivery agents.

### 3.8. In Vitro Digestion Characteristics

The data presented in Table 3 offers insights into the proportion of digestible and resistant starch in kudzu starch gel when exposed to different ethanol concentrations and durations of exposure. The data reveals a consistent pattern: as the concentration of ethanol increases, there was a commensurate decrease in the percentage of digestible starch for every time interval. Predictably, control samples consistently exhibited the highest digestibility percentages (97.11–98.15%). Moreover, there was a time-dependent decrease in digestibility within each ethanol concentration, suggesting that both ethanol’s presence and exposure duration critically influence the gel’s digestibility [35]. At a 2 h exposure, the control had a digestibility of 98.15%. In contrast, gels exposed to 100% ethanol offered a digestibility of 92.19%. This marked difference (*p* < 0.05) underscores ethanol’s potency in curbing starch gel digestibility, potentially due to ethanol’s interference with starch molecular structures [8,31].

The analysis of the residual undigested fraction (resistant starch) results revealed that with rising ethanol concentration comes an increase in the resistant starch. This trend holds true across all time intervals. Typically, the control for a 2 h exposure had a resistant starch level of 1.87%, while the sample at 80% ethanol records 7.92%. The data ranges from the control’s 1.87% at 2 h to a peak of 8.73% with 100% ethanol at 6 h. Drawing parallels with the digestible starch data, it is clear there was an inverse correlation (r = −0.845). The elevated resistant starch in the presence of ethanol might be a result of the immediate recrystallization of amylose, forming structures less accessible to enzymatic action [8,26,32,40]. Furthermore, the impediments caused by disruptions and subsequent rearrangements occurring within the starch matrix can hinder the penetration of enzymes. This phenomenon has been observed in other polysaccharides treated with ethanol, particularly after the drying process, as discussed by Sun et al. [2].

The residual resistant starch after 242 min. of oral, gastric, and intestinal digestion suggests relics of nondigestible portions of kudzu starch gels [42]. The proportion, however, improved with ethanol exposure over time. This finding reflects studies [28,35] positing that solvents, including ethanol, can alter starch structures, rendering them more resistant to enzymatic degradation. This observed ethanol modulating effect is desirable due to the established beneficial health significance of resistant starches including improved gut health, blood sugar regulation, satiety, and potential disease risk reduction [43,44,45]. Based on the findings, the increased concentration of resistant starch in the kudzu starch gel exposed to ethanol indicates a potential enhancement in its health-promoting properties when compared to the untreated kudzu starch gel. In line with the health implications, ethanol is volatile and rapidly evaporates after treatment, rendering its residue in the final product negligible [46]. The potential ethanol remnant in our kudzu starch is therefore innocuous, comparable to what is found in everyday foods, and would not result in any detrimental health effects upon consumption or as a component of food. In essence, we opine that our study resonates with the existing literature regarding the nuanced interplay between ethanol, starch gel mechanical properties, crystallinity, and digestibility.

## 4. Conclusions

Our research highlighted the profound influence of ethanol concentration and exposure time on kudzu starch gels. At the lowest ethanol concentrations, gel strength was still enhanced, attributed to a water–ethanol dehydration mechanism. The decreased T_c_-T_o_ with increased ethanol suggests faster thermal transitions. Ethanol reduced syneresis, which corresponded with changes in gel size and color. The starch gels displayed a shear-thinning behavior validated by the power law model, with R^2^ > 0.95. Furthermore, the higher G′ over G′′ coupled with Tan δ values emphasized the gels’ elasticity, modified slightly by ethanol.

SEM images reveal matrix densification upon ethanol treatment, an essential characteristic for efficient absorption and encapsulation of bioactive constituents. Furthermore, the gels’ crystalline characteristics and FTIR spectra remained largely unchanged post ethanol exposure, indicating the absence of detrimental chemical changes due to ethanol. Digestibility tests revealed a shift from digestible starch to resistant starch, providing potential benefits for gut health, diabetes management, and metabolic syndrome prevention. In conclusion, our results emphasize ethanol’s transformative impact on kudzu starch gel’s mechanical, rheological, and digestibility properties, suggesting opportunities to optimize starch-based products. The enhanced robustness of the ethanol-treated kudzu starch gels aligns well with gel-based desserts, confectionery needs, beverages, and nutritional pharmaceuticals, where a consistent texture is essential.

## Figures and Tables

**Table 1:** Constituent and volume of simulated digestion stock solutions.

Simulated Fluids	SSF (Mouth)		SGF (Stomach)		SIF (Intestine)	
Constituent	(mM)	mL	(mM)	mL	(mM)	mL
KCl	15.1	15.1	6.9	6.9	6.8	6.8
KH_2_PO_4_	3.7	3.7	0.9	0.9	0.8	0.8
NaHCO_3_	13.6	6.8	25	12.5	85	42.5
NaCl	-	-	47.2	11.8	38.4	9.6
MgCl_2_(H_2_O)_6_	0.15	0.5	0.12	0.4	0.33	1.1
(NH_4_)_2_CO_3_	0.06	0.06	0.5	0.5	-	-
HCl	1.1	0.09	15.6	1.3	8.4	0.7
CaCl_2_(H_2_O)_2_	1.5	0.025	0.15	0.005	0.6	0.04

SSF: Simulated Salivary Fluid; SGF: Simulated Gastric Fluid; SIF: Simulated Intestinal Fluid. -: excluded from the simulated fluid. Adopted from INFOGEST [21].

**Table 2:** The mechanical characteristics and fitted rheological parameter of kudzu starch gel exposed to different ethanol concentrations and time.

Samples		Textural Characteristics				Fitted Rheological Parameters		
EC	ET	Hardness (g)	Springiness	Resilience	Chewiness (g)	*k*	*n*	R^2^
0%		62.22 ± 1.52 ^a^	0.54 ± 0.02 ^a^	61.31 ± 1.61 ^a^	52.81 ± 1.98 ^a^	1055 ± 73.01 ^g^	−0.63 ± 0.02 ^b^	0.974 ^d^
30%	2 h	154.55 ± 2.01 ^b^	0.61 ± 0.02 ^b^	73.58 ± 3.15 ^b^	57.08 ± 0.99 ^b^	2235 ± 150 ^c^	−0.73 ± 0.03 ^ef^	0.963 ^g^
60%		153.52 ± 1.52 ^b^	0.73 ± 0.04 ^c^	73.95 ± 1.37 ^b^	70.52 ± 1.22 ^ed^	2145 ± 105 ^cd^	−0.71 ± 0.02 ^e^	0.978 ^bc^
80%		165.08 ± 2.23 ^c^	0.73 ± 0.01 ^c^	75.41 ± 2.11 ^c^	71.18 ± 3.01 ^e^	1999 ± 137 ^e^	−0.69 ± 0.02 ^d^	0.973 ^d^
100%		168.62 ± 3.11 ^cd^	0.79 ± 0.03 ^d^	74.82 ± 1.37 ^bc^	73.02 ± 1.86 ^ef^	2867 ± 124 ^b^	−0.89 ± 0.02 ^g^	0.990 ^a^
30%	4 h	157.25 ± 1.01 ^b^	0.63 ± 0.02 ^b^	74.58 ± 2.05 ^bc^	58.21 ± 0.98 ^b^	661 ± 30 ^h^	−0.58 ± 0.01 ^a^	0.977 ^c^
60%		164.05 ± 1.61 ^c^	0.79 ± 0.04 ^d^	78.23 ± 1.66 ^d^	69.98 ± 1.81 ^d^	1932 ± 205 ^e^	−0.66 ± 0.04 ^c^	0.965 ^f^
80%		169.29 ± 2.41 ^d^	0.81 ± 0.01 ^de^	81.16 ± 1.31 ^f^	75.22 ± 3.44 ^f^	3698 ± 55 ^a^	−1.62 ± 0.07 ^h^	0.955 ^h^
100%		177.37 ± 1.22 ^f^	0.98 ± 0.03 ^b^	79.15 ± 2.18 ^e^	79.11 ± 1.61 ^g^	1358 ± 65 ^f^	−0.67 ± 0.01 ^cd^	0.979 ^bc^
30%	6 h	155.08 ± 1.05 ^b^	0.71 ± 0.02 ^c^	80.82 ± 3.25 ^ef^	63.71 ± 1.00 ^c^	2149 ± 104 ^c^	−0.70 ± 0.02 ^de^	0.980 ^b^
60%		169.53 ± 1.84 ^d^	0.83 ± 0.04 ^e^	85.23 ± 1.63 ^g^	69.45 ± 1.35 ^d^	1991 ± 136 ^e^	−0.69 ± 0.02 ^d^	0.982 ^ab^
80%		171.51 ± 2.23 ^e^	0.97 ± 0.01 ^f^	89.91 ± 2.07 ^h^	79.89 ± 2.03 ^g^	2102 ± 121 ^d^	−0.62 ± 0.02 ^b^	0.971 ^e^
100%		178.82 ± 1.69 ^f^	1.01 ± 0.03 ^g^	91.15 ± 2.23 ^h^	79.89 ± 1.55 ^c^	1045 ± 74 ^g^	−0.65 ± 0.02 ^c^	0.966 ^f^

Data are presented as mean ± standard deviation. EC—Ethanol Concentration, ET—Exposure Time, *k*—consistency index, *n*—flow behavior index. Results along the same column with different superscript letters are significantly different at *p* < 0.05 (*n* = 3).

**Table 3:** Thermal transition, digestibility, syneresis, and relative crystallinity of kudzu starch gel exposed to ethanol at different concentrations and time.

Samples		Thermal Characteristics					Digestibility		Others	
EC	ET	T_o_ (°C)	T_p_ (°C)	T_c_ (°C)	T_c_-T_o_ (°C)	ΔH_g_ (J/g)	DS %	RS %	S %	RC %
(0%)		45.32 ± 0.1 ^b^	53.92 ± 0.3 ^b^	66.92 ± 0.3 ^b^	8.83 ± 0.1 ^a^	1.02 ± 0.3 ^f^	98.15 ± 0.43 ^a^	1.87 ± 0.12 ^h^	11.00 ± 0.1 ^a^	4.73 ± 0.3 ^j^
30%	2 h	45.32 ± 0.2 ^b^	58.92 ± 0.3 ^b^	64.92 ± 0.3 ^b^	7.83 ± 0.1 ^a^	2.92 ± 0.3 ^b^	95.08 ± 0.65 ^b^	4.92 ± 0.19 ^g^	11.03 ± 0.0 ^a^	5.21 ± 0.1 ^i^
60%		45.80 ± 0.4 ^b^	60.95 ± 0.1 ^b^	65.95 ± 0.1 ^b^	7.01 ± 0.6 ^c^	2.81 ± 0.1 ^ab^	93.75 ± 0.83 ^c^	6.25 ± 0.23 ^d^	10.04 ± 0.3 ^b^	5.93 ± 0.4 ^g^
80%		46.41 ± 0.5 ^a^	61.89 ± 0.1 ^b^	65.89 ± 0.1 ^b^	7.11 ± 0.3 ^b^	2.79 ± 0.1 ^b^	92.67 ± 0.36 ^c^	7.92 ± 0.52 ^bc^	9.60 ± 0.1 ^c^	6.82 ± 0.2 ^e^
100%		42.09 ± 0.7 ^d^	50.97 ± 0.3 ^b^	63.97 ± 0.3 ^b^	7.28 ± 0.2 ^c^	2.97 ± 0.3 ^a^	92.19 ± 0.88 ^cd^	7.81 ± 0.18 ^bc^	7.12 ± 0.2 ^g^	7.25 ± 0.3 ^c^
30%	4 h	43.12 ± 0.4 ^c^	59.53 ± 0.2 ^b^	65.53 ± 0.2 ^b^	6.54 ± 0.5 ^b^	2.53 ± 0.2 ^c^	94.92 ± 0.91 ^b^	5.09 ± 0.21 ^e^	8.41 ± 0.1 ^d^	5.74.7 ± 0.1 ^h^
60%		45.32 ± 0.1 ^b^	52.92 ± 0.3 ^b^	62.92 ± 0.3 ^b^	6.83 ± 0.1 ^a^	2.12 ± 0.3 ^b^	93.61 ± 0.57 ^c^	6.39 ± 0.47 ^d^	7.92 ± 0.1 ^e^	6.23 ± 0.2 ^f^
80%		43.10 ± 0.3 ^c^	62.95 ± 0.1 ^b^	69.95 ± 0.1 ^b^	6.01 ± 0.6 ^c^	2.25 ± 0.1 ^cd^	92.31 ± 0.72 ^d^	7.69 ± 0.29 ^c^	7.43 ± 0.4 ^f^	7.11 ± 0.0 ^d^
100%		41.41 ± 0.5 ^e^	59.89 ± 0.1 ^b^	63.89 ± 0.1 ^b^	5.11 ± 0.3 ^b^	2.09 ± 0.1 ^f^	91.93 ± 0.73 ^e^	8.05 ± 0.34 ^b^	6.55 ± 0.0 ^i^	7.59 ± 0.1 ^b^
30%	6 h	42.09 ± 0.5 ^d^	58.97 ± 0.3 ^b^	63.97 ± 0.3 ^b^	6.28 ± 0.2 ^c^	1.98 ± 0.3 ^b^	94.96 ± 0.49 ^b^	5.03 ± 0.35 ^f^	7.31 ± 0.2 ^fg^	5.92 ± 0.0 ^f^
60%		41.12 ± 0.2 ^e^	61.53 ± 0.2 ^b^	64.53 ± 0.2 ^b^	5.82 ± 0.5 ^b^	2.35 ± 0.2 ^d^	92.91 ± 0.99 ^c^	7.07 ± 0.52 ^c^	7.19 ± 0.1 ^g^	6.74 ± 0.0 ^e^
80%		43.09 ± 0.3 ^c^	63.97 ± 0.3 ^b^	11.97 ± 0.3 ^b^	6.28 ± 0.2 ^c^	2.17 ± 0.3 ^e^	91.87 ± 0.94 ^d^	8.14 ± 0.33 ^a^	6.72 ± 0.3 ^h^	7.51 ± 0.2 ^b^
100%		41.12 ± 0.4 ^e^	53.53 ± 0.2 ^b^	16.53 ± 0.2 ^b^	6.52 ± 0.5 ^b^	2.83 ± 0.2 ^b^	91.27 ± 0.81 ^d^	8.73 ± 0.25 ^a^	5.96 ± 0.3 ^j^	7.90 ± 0.1 ^a^

Data are presented as mean ± standard deviation. Results along the same column with different superscript letters are significantly different at *p* < 0.05 (*n* = 3). EC = Ethanol Concentration, ET = Exposure Time, S = Syneresis, RC = Relative Crystallinity, DS = Digestible Starch, RS = Resistant Starch, Others (S and RC) = characteristics that had only one parameter evaluated.

**Table 4:** Pearson correlation analysis of gel mechanical properties, syneresis, relative crystallinity, size, digestibility, and color parameters.

	Syneresis	RC	Hardness	Springiness	Resilience	Chewiness	Size	DS	RS	L*	a*	b*
Syneresis	1											
RC	0.725	1										
Hardness	0.689	0.537	1									
Springiness	0.692 *	0.672	0.820 *	1								
Resilience	0.753 *	−0.599	0.863 *	0.795 *	1							
Chewiness	0.521	0.659	0.699	0.574	0.894 *	1						
Size	0.746	−0.371	−0.592	0.498	−0.569	0.632	1					
DS	−0.649	−0.823 *	−0.516	0.564	0.628	−0.748	0.297	1				
RS	0.853 *	0.795 *	0.647	0.594	0.774 *	0.539	0.211	−0.845 *	1			
L*	0.572	0.439	0.154	0.473 *	−0.367	0.255	0.527	0.319	0.198	1		
a*	−0.434	0.255	0.316	−0.259	0.401	0.374 *	0.498	0.225	0.254	−0.789 *	1	
b*	−0.413	0.398 *	0.299	0.325	0.384	0.296	0.596	0.497	0.352	0.522	0.658 *	1

RC—Relative Crystallinity, DS—Digestible Starch, RS—Resistant Starch, L*—Lightness, a*—Redness, b*—Yellowness. Values with asterisk showed statistical significance at *p* < 0.05.

## Data Availability

The data used to support the findings of this study can be made available by the corresponding author upon request.

## References

[1] Ahmed E.M. (2015). Hydrogel: Preparation, characterization, and applications: A review. J. Adv. Res..

[2] Sun Q., Li P., Li Y., Ji N., Dai L., Xiong L., Sun Q. (2020). Rapid production of corn starch gels with high mechanical properties through alcohol soaking. Int. J. Biol. Macromol..

[3] Klein M., Poverenov E. (2020). Natural biopolymer-based hydrogels for use in food and agriculture. J. Sci. Food Agric..

[4] Nepovinnykh N.V., Kliukina O.N., Ptichkina N.M., Bostan A. (2019). Hydrogel based dessert of low calorie content. Food Hydrocoll..

[5] Li X., Sun Q., Li Q., Kawazoe N., Chen G. (2018). Functional hydrogels with tunable structures and properties for tissue engineering applications. Front. Chem..

[6] Lin X., Zhao X., Xu C., Wang L., Xia Y. (2022). Progress in the mechanical enhancement of hydrogels: Fabrication strategies and underlying mechanisms. J. Polym. Sci..

[7] Liu F., Wu D., Hong W. (2023). Mechanism study on mechanical properties of physical–chemical hybrid hydrogels by coarse-grained molecular dynamics simulations. ACS Appl. Polym. Mater..

[8] Zheng Q., Liu Z., Liang X., Zhou Y., Zhao G. (2023). Insights into network rearrangement of konjac glucomannan gel induced by post-gelation soaking. Food Hydrocoll..

[9] Sun Y., Li F., Luan Y., Li P., Dong X., Chen M., Dai L., Sun Q. (2021). Food hydrocolloids gelatinization, pasting, and rheological properties of pea starch in alcohol solution. Food Hydrocoll..

[10] Arabi S.H., Haselberger D., Hinderberger D. (2020). The effect of ethanol on gelation, nanoscopic, and macroscopic properties of serum albumin hydrogels. Molecules.

[11] Van Vlierberghe S., Dubruel P., Schacht E. (2011). Biopolymer-based hydrogels as scaffolds for tissue engineering applications: A review. Biomacromolecules.

[12] Reddy C.K., Luan F., Xu B. (2017). Morphology, crystallinity, pasting, thermal and quality characteristics of starches from adzuki bean (Vigna angularis L.) and edible kudzu (Pueraria thomsonii Benth). Int. J. Biol. Macromol..

[13] Li H., Cui B., Janaswamy S., Guo L. (2019). Structural and functional modifications of kudzu starch modified by branching enzyme. Int. J. Food Prop..

[14] Zhao Y., Zhu X., Fang Y. (2021). Structure, properties and applications of kudzu starch. Food Hydrocoll..

[15] Tovar J., Melito C., Herrera E., Rascón A., Pérez E. (2002). Resistant starch formation does not parallel syneresis tendency in different starch gels. Food Chem..

[16] Ekumah J.-N., Ma Y., Akpabli-Tsigbe N.D., Kwaw E., Jie H., Quaisie J., Manqing X., Johnson Nkuma N.A. (2021). Effect of selenium supplementation on yeast growth, fermentation efficiency, phytochemical and antioxidant activities of mulberry wine. LWT.

[17] Adewale F.J., Lucky A.P., Oluwabunmi A.P., Boluwaji E.F. (2017). Selecting the most appropriate model for rheological characterization of synthetic based drilling mud. Int. J. Appl. Eng. Res..

[18] Ahmad M., Mudgil P., Gani A., Hamed F., Masoodi F.A., Maqsood S. (2019). Nano-encapsulation of catechin in starch nanoparticles: Characterization, release behavior and bioactivity retention during simulated in-vitro digestion. Food Chem..

[19] Zhang J., Yu P., Fan L., Sun Y. (2021). Effects of ultrasound treatment on the starch properties and oil absorption of potato chips. Ultrason. Sonochem..

[20] Wu J., Xu S., Huang Y., Zhang X., Liu Y., Wang H., Zhong Y., Bai L., Liu C. (2022). Prevents kudzu starch from agglomeration during rapid pasting with hot water by a non-destructive superheated steam treatment. Food Chem..

[21] Brodkorb A., Egger L., Alminger M., Alvito P., Assunção R., Ballance S., Bohn T., Bourlieu-Lacanal C., Boutrou R., Carrière F. (2019). INFOGEST static in vitro simulation of gastrointestinal food digestion. Nat. Protoc..

[22] Ahearne M., Yang Y., Liu K. (2008). Mechanical characterisation of hydrogels for tissue engineering applications. Top. Tissue Eng..

[23] Alam F., Hasnain A. (2009). Studies on swelling and solubility of modified starch from taro (*Colocasia esculenta*): Effect of pH and temperature. Agric. Conspec. Sci..

[24] Mohamed A., Hussain S., Alamri M.S., Ibraheem M.A., Abdo Qasem A.A., Ababtain I.A. (2022). Physicochemical Properties of Starch Binary Mixtures with Cordia and Ziziphus Gums. Processes.

[25] Akonor P.T., Tortoe C., Oduro-Yeboah C., Saka E.A., Ewool J. (2019). Physicochemical, microstructural, and rheological characterization of tigernut (*Cyperus esculentus*) Starch. Int. J. Food Sci..

[26] Mohamed I.O. (2021). Effects of processing and additives on starch physicochemical and digestibility properties. Carbohydr. Polym. Technol. Appl..

[27] Mohd Shukri A., Cheng L.H. (2023). The Properties of Different Starches under the Influence of Glucono-Delta-Lactone at Different Concentrations. Foods.

[28] Song Q., Wu L., Li S., Zhao G., Cheng Y., Zhou Y. (2022). Aggregation of konjac glucomannan by ethanol under low-alkali treatment. Food Chem. X.

[29] Roux D.C.D., Jeacomine I., Maîtrejean G., Caton F., Rinaudo M. (2023). Characterization of Agarose Gels in Solvent and Non-Solvent Media. Polymers.

[30] Cassanelli M., Norton I., Mills T. (2017). Effect of alcohols on gellan gum gel structure: Bridging the molecular level and the three-dimensional network. Food Struct..

[31] Subrahmanyam R., Gurikov P., Dieringer P., Sun M., Smirnova I. (2015). On the road to biopolymer aerogels—Dealing with the solvent. Gels.

[32] Landim Parente G.D., de Almeida Macêdo F., Melo Diniz N.C., da Conceição M.M., Ubbink J., Mattos Braga A.L. (2021). Observations on the formation and textural properties of “tapiocas”, a traditional cassava-based food from the Northeast of Brazil. Int. J. Gastron. Food Sci..

[33] Aleixandre A., Benavent-Gil Y., Moreira R., Rosell C.M. (2021). Food hydrocolloids in vitro digestibility of gels from different starches: Relationship between kinetic parameters and microstructure. Food Hydrocoll..

[34] Benavent-Gil Y., Rosell C.M. (2017). Comparison of porous starches obtained from different enzyme types and levels. Carbohydr. Polym..

[35] Jiang Q., Gao W., Li X., Zhang J., Huang L. (2011). Effect of acid-ethanol on the physicochemical properties of Dioscorea opposita Thunb. and Pueraria thomsonii Benth. starches. Starch-Stärke.

[36] Chen B., Dang L., Zhang X., Fang W., Hou M., Liu T., Wang Z. (2017). Physicochemical properties and micro-structural characteristics in starch from kudzu root as affected by cross-linking. Food Chem..

[37] Li X., Wang C., Lu F., Zhang L., Yang Q., Mu J., Li X. (2015). Physicochemical properties of corn starch isolated by acid liquid and l-cysteine. Food Hydrocoll..

[38] Shang X., Wang Q., Li J., Zhang G., Zhang J., Liu P., Wang L. (2021). Double-network hydrogels with superior self-healing properties using starch reinforcing strategy. Carbohydr. Polym..

[39] Vamadevan V., Bertoft E. (2015). Structure-function relationships of starch components. Starch-Stärke.

[40] Chang R., Ji N., Li M., Qiu L., Sun C., Bian X., Qiu H., Xiong L., Sun Q. (2019). Green preparation and characterization of starch nanoparticles using a vacuum cold plasma process combined with ultrasonication treatment. Ultrason. Sonochem..

[41] Cornejo-Ramírez Y.I., Martínez-Cruz O., Del Toro-Sánchez C.L., Wong-Corral F.J., Borboa-Flores J., Cinco-Moroyoqui F.J. (2018). The structural characteristics of starches and their functional properties. CYTA J. Food.

[42] Song X., Dong H., Zang Z., Wu W., Zhu W., Zhang H., Guan Y. (2021). Kudzu resistant starch: An effective regulator of type 2 diabetes mellitus. Oxid. Med. Cell. Longev..

[43] Shah A., Masoodi F.A., Gani A., Ashwar B.A. (2016). In-vitro digestibility, rheology, structure, and functionality of RS3 from oat starch. Food Chem..

[44] Yu M., Shin M. (2015). Improving gel formation of rice starch added with cross-linked resistant starch prepared from rice starch. Starch-Stärke.

[45] Campbell J. (2010). High-Throughput Assessment of Bacterial Growth Inhibition by Optical Density Measurements. Curr. Protoc. Chem. Biol..

[46] Tulashie S.K., Appiah A.P., Torku G.D., Darko A.Y., Wiredu A. (2017). Determination of methanol and ethanol concentrations in local and foreign alcoholic drinks and food products (Banku, Ga kenkey, Fante kenkey and Hausa koko) in Ghana. Int. J. Food Contam..

